# To the “Dental Review”

**Published:** 1891-10

**Authors:** 


### TO THE "DENTAL REVIEW."

Are we correct in presuming that the failure to give the Journal credit for its "List of Houses at Saratoga Springs, N. Y.," copied in the July number of the *Dental Review*, was one of those unintentional errors that will occasionally annoy the most conscientious editor?

